# From Willingness to Readiness: Caregiver Activation for Cancer Care in Singapore

**DOI:** 10.3390/ijerph23050661

**Published:** 2026-05-15

**Authors:** Safora Johansen, Melissa Gaik Ming Ooi, Alice F. S. Chua

**Affiliations:** 1 Faculty of Health Sciences, Oslo Metropolitan University, 0167 Oslo, Norway; 2 Health and Social Sciences Cluster, Singapore Institute of Technology, Singapore 828608, Singapore; 3 Cancer Clinic, Oslo University Hospital, 0318 Oslo, Norway; 4 Department of Medicine, Yong Loo Lin School of Medicine (YLLSOM), National University of Singapore, Singapore 119228, Singapore; 5 National University Cancer Institute Singapore, National University Hospital, Singapore 117597, Singapore; 6 Department of Nursing Administration (DNA), National Cancer Centre Singapore, Singapore 168583, Singapore

**Keywords:** caregiver activation, CG-PAM, older adults, cancer, Singapore, self-management, outpatient care, caregiver support

## Abstract

**Highlights:**

**Public health relevance—How does this work relate to a public health issue?**

- Caregiver activation has the potential to improve health systems/healthcare, and this is the first study to examine caregiver activation in Singapore.
- This study may be a first step towards developing pathways to support caregiver activation.

**Public health significance—Why is this work of significance to public health?**

- This study identifies caregiver activation, a modifiable determinant of outpatient cancer outcomes, by characterizing caregivers’ knowledge, skills, confidence, and stress resilience in Singapore. It advances understanding of caregiver activation in an Asian setting, where such work has been limited.
- These findings provide insight into the current status of caregivers in an Asian context and will inform future research to improve caregiving.

**Public health implications—What are the key implications or messages for practitioners, policy makers and/or researchers in public health?**

- Treat caregiver activation as a routine, standardized metric across cancer care—integrate screening (e.g., CG-PAM) at key touchpoints, use activation (not demographics) to guide support, and continuously assess its validity and responsiveness across populations and settings.
- Develop, implement, and scale activation-tailored supports that strengthen stress resilience and sustainment (education, coaching, respite, digital boosters) and rigorously evaluate their clinical, equity, and economic impacts to inform sustainable models that reduce avoidable healthcare utilization.

**Abstract:**

Background: Family caregivers are essential partners in the outpatient care of older adults with cancer, yet the knowledge, skills, and confidence, collectively, of caregiver activation are not well characterized in Asian settings. Understanding activation can inform tailored support to sustain effective caregiving. Accordingly, this study assessed the overall activation levels among cancer caregivers in the three most common cancer diagnoses in Singapore by using the Caregiver-Patient Activation Measure (CG-PAM). Methods: A total of 103 informal caregivers of patients ≥60 years (lung, GI, or myeloma) at Singapore’s largest public cancer hospitals completed the 13-item CG-PAM, scored 0–100 and classified into four activation levels. Descriptive statistics summarized characteristics and CG-PAM responses, and logistic regression analyses assessed the degree of activation for individual demographic and other characteristics (*p* < 0.05). Results: Caregivers showed moderate–high activation (mean 65.9 ± 16.1) and the following levels: L1, 4.9%; L2, 23.3%; L3, 38.8%; L4, 31.1%. They strongly endorsed personal responsibility and active engagement, reporting confidence in key self-management tasks, but struggled to sustain behaviors under stress. Activation was not significantly associated with demographic factors or any other measured characteristics. Conclusions: Caregivers of older adults with cancer in Singapore included in this study showed moderate–high activation and strong self-management confidence but struggled to sustain behaviors under stress. Routine activation assessment and tailored support (education, coaching) could strengthen outpatient care. Longitudinal and interventional research, alongside follow-up interviews, is needed to identify unmet needs, existing support systems, and inform scalable, sustainable models.

## 1. Introduction

Family and informal caregivers play a pivotal role in the care of older adults with cancer, often coordinating appointments, managing complex treatment regimens, monitoring symptoms, and supporting lifestyle changes over prolonged periods [1]. As cancer care becomes increasingly outpatient and decentralized, the capacity of caregivers to engage, learn, and take effective action, commonly referred to as caregiver activation, has gained prominence as a determinant of patient and caregiver outcomes. Caregiver activation encompasses knowledge, skills, confidence, and self-efficacy to navigate health information, communicate with clinicians, and carry out day-to-day self-management tasks that complement clinical care [2,3,4,5,6,7,8].

Within oncology and geriatric care, caregiver activation is particularly salient. Older adults frequently present with multimorbidity, functional limitations, and unique social needs, placing added demands on caregivers’ problem-solving and decision-making [9]. Although activation has been studied among patients, less is known about activation among caregivers, especially in Asian contexts where cultural expectations, family structures, and health system factors may shape caregiving roles and resources differently than in Western settings. Understanding activation in Singapore, where public hospitals provide the bulk of cancer services, can inform tailored support strategies that are culturally sensitive and aligned with local care pathways [10].

Caregiver activation is the knowledge, skills, and confidence of the informal caregiver providing care for the patient [2]. It empowers caregivers to manage care, addressing healthcare needs, improvement of patients’ quality of life, and patient satisfaction while reducing costs [2,3]. Strategies to support caregiver activation include collaborative processes for shared decision-making, measurable and achievable goal setting, and motivational interviewing with reflective listening [4]. Studies [5,7,8] report increased caregiving hours, high caregiver burden, and increased anxiety and/or depression among Singaporean caregivers, suggesting the need for additional support and screening for distress.

Caregiver activation refers to the extent of a caregiver’s skills, knowledge, and confidence in providing care [3,11]. Evidence confirms that higher caregiver activation, defined as caregivers’ knowledge, skills, and confidence [11], improves quality of life for both patients and caregivers [3,11,12]. Validated instruments such as the Caregiver Activation Measure (CG-PAM) [3,11,13] enable standardized assessment of activation, segmenting caregivers into four levels that reflect increasing knowledge, skills, and confidence. These levels can guide the design and targeting of interventions, helping clinicians and health systems identify caregivers who may benefit from additional education, coaching, or community resources to sustain effective caregiving over time [11,12,13,14,15].

This study was conducted in partnership with Singapore’s largest public institutions providing cancer care and focuses on caregivers of older adults with lung, gastrointestinal, or myeloma cancers. Our objectives were to assess the overall activation levels among cancer caregivers in the three most common cancer diagnoses in Singapore by using the CG-PAM. This assessment describes caregivers’ self-management attitudes and skills and examines the degree of activation for individual demographic caregiving characteristics. By characterizing activation profiles in this population, we aim to generate evidence that can inform practical, scalable strategies to support caregivers and ultimately enhance the quality and continuity of cancer care for older adults.

## 2. Methods

### 2.1. Study Design and Setting

This exploratory, cross-sectional quantitative study, conducted in partnership with Singapore’s largest public hospitals providing cancer care, assessed caregiver activation among caregivers of older adults with cancer.

### 2.2. Recruitment and Data Collection

Using convenience sampling strategy, cancer patients aged 60 years or older with lung, gastrointestinal (GI), or myeloma cancer stage I–III who were undergoing cancer treatment were approached during routine follow-up visits with their oncologist or oncology nurse, both of whom were members of the research team. The purpose of these follow-up visits was to monitor well-being and manage symptoms. Patients received a brief description of the study and were asked whether they had a primary informal caregiver who might be interested in participating. They were given an information letter with a research contact and a one-page, 13-statement CG-PAM questionnaire [3,11,13] to share with their caregivers. Most patients attended appointments with their caregivers; refusals were thus recorded from both patients and caregivers at the hospital. Caregivers who wished to participate provided informed consent by completing the questionnaire and returning it to the research team member at the hospital. Some caregivers contacted the designated research contact to clarify questions before deciding to participate. No data was collected on cancer treatment type. However, all patients received the same treatment regimen—chemotherapy and radiation. Hospitalized patients and those in the terminal phase were not approached. Recruitment occurred between September 2025 and December 2025. Lung and gastrointestinal cancers are the most common solid cancers, and myeloma is a common hematological cancer in Singapore. These cancers also have a long treatment duration necessitating prolonged caregiver involvement.

Caregiver activation was assessed with the 13-item Caregiver Patient Activation Measure ([3,11,13] Appendix A). The CG-PAM evaluates caregivers’ knowledge and performance related to their responsibilities and the care recipient’s healthcare needs to produce a composite activation score. That score is then converted into an overall activation level from 1 to 4:

Level 1: May not yet believe they play a role in managing the patient’s health or that their role is important (score ≤ 47).

Level 2: Lacks confidence and knowledge to take action on the patient’s behalf (score 47.1–55.1).

Level 3: Beginning to take action and feeling confident they are in charge (score 55.2–67).

Level 4: Confident but may have difficulty maintaining their level of involvement over time (score ≥ 67.1).

The CG-PAM is self-administered and includes 13 items rated on a four-point Likert scale (1 = disagree strongly, 2 = disagree, 3 = agree, 4 = strongly agree), with an N/A option for items that do not apply. Raw CG-PAM data were scored by Insignia Health [3,11,13].

As part of the survey, participants provided demographic information and indicated whether they were interested in a follow-up interview to be included in a separate publication.

Inclusion criteria for cancer caregivers:

(1) Unpaid family member or close contact providing regular care to an older cancer patient (≥60 years).
(2) Relationship includes spouse, adult child (≥21 years), other relatives, or close friends.
(3) English-speaking caregiver of Asian descent (Chinese, Malay, or Indian ethnicity).
(4) Caregiver to a patient with lung, GI, or myeloma cancer who is not in terminal phase.
(5) Capable of and willing to provide informed consent.
(6) Able to understand the study objectives and complete an English-language self-report questionnaire.
(7) No history of severe psychiatric illness.

### 2.3. Data Analysis

Survey and demographic data were exported to IBM SPSS Statistics for Windows, version 31.0 (IBM Corp., Armonk, NY, USA, 2025), for analysis. Participant characteristics and study measures were summarized using descriptive statistics (frequencies, percentages, means, and standard deviations as appropriate). The population was divided into two groups: low activation (levels 1–2) and high activation (levels 2–3). This category was used as dependent variable. The degree of activation for individual demographic and other characteristics was assessed using logistic regression analyses (binary logistic). Statistical significance was set at *p* < 0.05. Odds ratio and 95% confidence interval (CI) were calculated.

### 2.4. Ethical Considerations

The study was approved by Ethics and Compliance Online System (ECOS Ref: 2025-0988) on 18 August 2025. All participants provided informed consent prior to participation. Participant confidentiality was maintained throughout recruitment, data collection, storage, and analysis. Informed consent was obtained by completing the survey.

## 3. Results

### 3.1. Sample Characteristics

**Figure 1:**
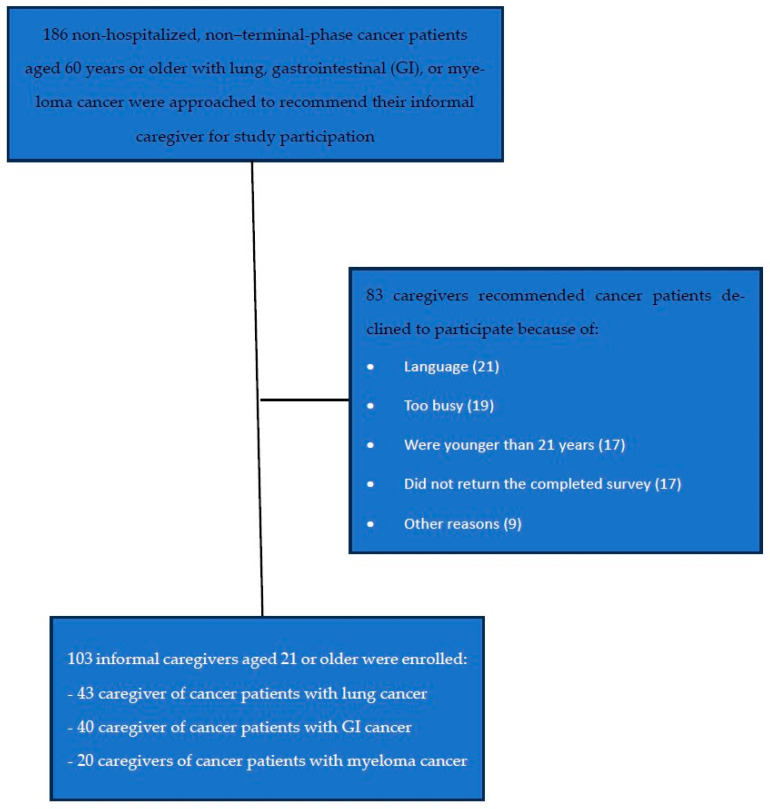
Flowchart of recruitment process.

Demographic characteristics are summarized in Table 1. A total of 103 cancer caregivers were recruited to participate in this study as reported in Figure 1 and completed the activation measurement survey (mean age, 54.6 years); 62.1% of these were female. Most identified as Chinese (69.9%).

Nearly half were spouses/partners of the patient (49.5%), followed by sons/daughters (30.1%). Most reported lower educational attainment (62.1%), and 70.9% were married. Regarding employment, 53.4% were employed full-time and 21.3% were retired. The duration of caregiving was most commonly 1–3 years (51.5%), followed by 3–6 years (18.4%). The vast majority were first-time caregivers (98.1%). Monthly household income varied, with 37.9% reporting less than Singapore Dollar (SGD) 2000 and 27.2% reporting more than SGD 8000.

### 3.2. Caregiver Activation

The Caregiver Activation Measure (CG-PAM) as reported in Table 2 showed a median score of 60.6 (mean, 65.9; SD, 16.1). Based on activation levels, 4.9% were Level 1 (may not believe their role is important), 23.3% were Level 2 (lacking knowledge and confidence), 38.8% were Level 3 (taking action and gaining control), and 31.1% were Level 4 (confident but may struggle to maintain behaviors over time). Activation varied modestly by cancer type.

### 3.3. Self-Management Attitudes and Skills

Detailed responses to 13 self-activation questions are reported in Appendix A. Caregivers strongly endorsed personal responsibility and active engagement in health: a total of 97.1% agreed or strongly agreed they are responsible for taking care of their health, and 99.0% agreed or strongly agreed that taking an active role is the most important factor affecting their health. Most expressed confidence in core self-management abilities. Specifically, 87.4% agreed or strongly agreed that they can help prevent or reduce health problems, and 98.1% felt able to judge when to seek medical care versus self-manage. Confidence in communicating concerns to clinicians was high for the majority. Many reported knowing what their prescribed medications do (77.6% agreed/strongly agreed; 24.3% were marked not applicable), being able to carry out home medical treatments (78.7% agreed/strongly agreed; 15.5% were marked not applicable), and understanding their health problems and causes (73.8% agreed/strongly agreed; 16.5% were marked not applicable). Knowledge of available treatments was somewhat lower but still common (68.0% agreed/strongly agreed; 16.5% not applicable). Most reported maintaining lifestyle changes such as healthy eating and exercise (89.3% agreed/strongly agreed), though maintaining these changes during stress was more challenging for some (88.3% agreed/strongly agreed; 10.7% disagreed). A large majority knew how to prevent problems with their health (92.2% agreed/strongly agreed) and felt able to work out solutions when new problems arise (84.4% agreed/strongly agreed).

### 3.4. Associations Between Caregiver Characteristics and Activation

In logistic regression analyses, caregiver activation was not significantly associated with any sociodemographic and characteristics of the included caregivers as reported in Table 3. These findings indicate no detectable differences in activation across the measured demographic and caregiving characteristics in this sample.

## 4. Discussions

As the first Asian study to measure caregiver activation using the CG-PAM, this work fills a key evidence gap by providing context-specific benchmarks for cancer caregiving in Singapore and comparable settings. In a cohort of 103 cancer caregivers included in this study, overall activation was moderate, with 39% at Level 3 and 31% at Level 4. Caregivers strongly endorsed self-management attitudes and skills, 97% agreed they are responsible for their health, and 99% affirmed that taking an active role is crucial; many reported confidence communicating with clinicians and performing home treatments. Notable gaps included limited understanding of what each medication does and difficulty sustaining lifestyle changes under stress. No statistical associations were found between activation and sociodemographic or caregiving factors (age, gender, relationship, income, care duration). Targeted approaches that build medication and treatment knowledge, support behavior maintenance under stress, and teach structured problem-solving can further enhance caregiver activation and help sustain the quality and continuity of cancer care for older adults.

### 4.1. Caregiver Activation in the Context of Outpatient Cancer Care

The median CG-PAM score in our cohort was 60.6, indicating higher activation than reported by Bakker et al. [3], with a median of 51 among caregivers of Dutch patients with a solid metastasized tumor providing informal care at 3 months of caregiving, but lower than the values reported by Mazanec et al. [16] at 4 weeks (63.4) and 6 weeks (70.5) in American caregivers of colorectal cancer patients. Whereas Mazanec et al. [16] observed a decline in activation with longer caregiving duration, our analysis found no significant association between length of caregiving and activation, with 15.5% of caregivers providing care for more than 6 years (*p* = 0.821). Furthermore, 70% of caregivers were categorized at Level 3 and 4, which represented their readiness to act, confidence in decision-making, and in active engagement with the healthcare team. The high level of caregiver activation observed in our study is notable, given that 62% self-reported lower educational attainment and at least half remained employed. This may reflect unique features of Singapore’s healthcare system that promote population health literacy, including universal access to secure personal health records, public health education talks, and annual patient education sessions organized by most cancer groups. Caregivers are frequently responsible for care coordination and supportive roles across the cancer trajectory and are integral to patient and caregiver well-being as confirmed by the existing literature [5,17,18]. Notably, none of the caregivers in this study were supporting patients in the terminal phase, a stage associated in the literature with declines in activation [19]. However, the absence of a dedicated caregiver health literacy program may underlie the insecurity caregivers reported regarding medications and their effects. Regardless of caregiver health literacy in Singapore or the sociodemographic characteristics reported in Table 1, our findings highlight strong family bonds that drive a willingness to provide care whenever needed, across ages and family circumstances. Despite overall high activation, approximately one quarter of caregivers were classified as Level 2, suggesting lower confidence and knowledge to act (Table 2). These findings indicate that caregivers may struggle with complex care demands, particularly during transitions from hospital-based to outpatient care [17,18], underscoring the need for early identification of caregivers who are willing but insufficiently prepared to enable targeted supportive strategies.

### 4.2. Self-Management Confidence and Sustainability Challenges

Caregivers reported high levels on self-reported confidence across core self-management domains for CG-PAM (Table 2), indicating personal commitment and accountability in the care of their loved ones. In addition, they demonstrated ability and confidence in early recognition for medical care needs, communication with healthcare professionals, and performing home-based medical care as indicated by the healthcare team, which is evident with the acquisition of experiential knowledge over time, especially in symptom monitoring and medication management [5,7]. A relative challenge for maintenance of caregivers’ lifestyle and caregiving behavior was flagged during periods of stress, during which caregivers indicated lower confidence levels in this domain (Table 2). Unsar and Ozdemir [20] observed a similar trend: as caregiving time increased, caregiving intensity, emotional burden, and illness-related uncertainty rose. This can obscure caregivers’ coping capacities and undermine the sustainability of long-term self-management. The importance of recognizing this distress and implementing proactive, cost-effective supportive interventions for caregivers is already highlighted by the existing literature [17].

### 4.3. Effect of Demographic Factors on Caregiver Activation

None of the caregiver sociodemographic or caregiving characteristics were significantly associated with higher activation (all *p*-values > 0.05; see Table 3 for odds ratios and 95% confidence intervals). One plausible explanation is that cultural expectations around caring for older family members are widely shared in Singapore, attenuating the influence of sociodemographic factors (e.g., age, education, income) on activation and thereby limiting variability. Another possibility is limited statistical power: with *n* = 103, wide confidence intervals indicate imprecision, and a substantially larger sample would be needed to detect modest effects with confidence. The lack of significant associations may also reflect the cohort’s generally high activation; highly activated caregivers often experience more positive outcomes, feel better prepared for care tasks, and maintain lower burden regardless of sociodemographic differences. Consistent with this, prior work has reported a direct association between higher activation and lower stress [20], and studies in advanced cancer and colorectal cancer survivors have found psychological readiness and perceived competence to exert greater influence than sociodemographic variables [3,5,21]. This readiness may be linked to Singapore’s family-oriented caregiving norms—particularly among spouses and adult children—and warrants further investigation.

### 4.4. Implications for Practice and Service Design

Findings from this exploratory study indicated the need for focused strategies to build confidence and management skills for caregivers with lower activation levels and concurrently built interventions to manage stress for caregivers with higher activation levels. The beneficial interventions might be focused on structured education, coaching, shared decision-making, and motivational interviewing; however, their effectiveness requires further evaluation [8]. With more healthcare systems transitioning healthcare from inpatient to outpatient settings, it is paramount to acknowledge the importance of caregivers’ roles as partners in care and develop a sustainable support structure to maintain the care quality and continuity of care [19,22]. Further research is underway to better understand caregivers’ unmet needs in Singapore.

### 4.5. Limitations

This quantitative study encompasses the utilization of a validated screening tool for evaluation of caregiver activation in the local context. Limitations include the lack of cancer patient disease history data and the recruitment of only English-speaking participants, which may limit generalizability. In addition, the small population included in this study is not homogenous in terms of age and the length of caregiving. We acknowledge the potential for response bias, whereby caregivers who completed the survey may be more activated than those who did not. Furthermore, the survey has not been culturally validated for local context. Nonetheless, the CG-PAM has been translated into multiple languages and used in diverse settings, suggesting broad applicability; its 13 items are intentionally general and designed to be relevant to caregivers across cultures.

Furthermore, other confounding variables of caregiver resilience and burden were not addressed in this study. In addition, disease stage and chemotherapy regimen were not collected, as the study focused on caregiver activation and experiences rather than clinical outcomes and in order to minimize participant burden in a survey-based design. However, we acknowledge that these factors may influence caregiving demands and could act as confounders. Future longitudinal and interventional research with clinical variables may provide more insights into the activation pattern and evaluation of activation-informed interventions on caregivers and its association with patient outcomes.

## 5. Conclusions

This is the first Asian study to measure caregiver activation using the CG-PAM. Across sociodemographic groups, caregivers maintained a high level of commitment to providing care. Caregivers responsible for caring for patients with cancer demonstrated overall moderate to high levels of activation as reflected by the CG-PAM and confidence in self-management scores in Singapore. However, significant gaps were identified in the maintenance of caregiver practices during stressful periods and among caregivers who possess lower confidence. Findings from this quantitative study highlight the need for further research to delineate caregivers’ unmet needs, inventory available support tools, and identify opportunities for early assessment and assistance; they also underscore the importance of longitudinal and intervention studies to evaluate and implement effective outpatient care systems.

## Figures and Tables

**Table 1:** Demographic characteristics of included cancer caregivers by cancer type.

Sample Characteristics	Sample Descriptions by Cancer Type			
	Myeloma (*n* = 20)	GI (*n* = 40)	Lung (*n* = 43)	Overall
**Relation of caregiver to the patient, *n* (%)** Spouse/partner Son/daughter Other	11 (55) 8 (40) 1 (5)	18 (45) 13 (32.5) 9 (22.5)	28 (65) 12 (28) 3 (7)	51 (49.5) 31 (30.1) 21 (20.4)
**Gender, *n* (%)** Male Female	7 (35) 13 (65)	13 (32.5) 27 (67.5)	19 (44.2) 24 (55.8)	39 (37.9) 64 (62.1)
**Age (years), mean (min, max), (SD)**	53.8, (32, 73), ±13.1	52.1, (29, 80), ±14.1	57.3, (21, 77), ±13.1	54.6 (21, 80), ±13.6
**Ethnicity, *n* (%)** Chinese Indian Malay Others	12 (60) 2 (10) 5 (25) 1 (5)	22 (55) 2 (5) 11 (27.5) 5 (12.5)	38 (88.4) 1 (2.3) 3 (7.0) 1 (2.3)	72 (69.9) 5 (4.9) 19 (18.4) 7 (6.8)
**Education, *n* (%)** Lower Higher (bachelor’s, master’s, or PhD)	10 (50) 10 (50)	28 (70) 12 (30)	26 (60.5) 17 (39.5)	64.0 (62.1) 39 (37.9)
**Employment status, *n* (%)** Full-time worker Part-time worker Retired Unemployed	8 (40) 2 (10) 4 (20) 6 (30)	22 (55) 4 (10) 9 (22.5) 5 (12.5)	22 (51.17) 6 (13.95) 11 (25.58) 4 (9.3)	55 (53.4) 12 (11.7) 22 (21.3) 14 (13.6)
**Civil status, *n* (%)** Married Single Divorced Widow	15 (75) 4 (20) 1 (5) 0	22 (55) 16 (40) 1 (2.5) 1 (2.5)	36 (83.7) 5 (11.6) 0 2 (4.7)	73 (70.9) 25 (24.3) 2 (1.9) 3 (2.9)
**Length of the** **provided care, *n* (%)** <1 year 1–3 years 3–6 years >6 years	5 (25) 8 (40) 3 (15) 4 (20)	4 (10) 22 (55) 7 (17.5) 7 (17.5)	6 (13.95) 23 (53.49) 9 (20.93) 5 (11.62)	15 (14.6) 53 (51.5) 19 (18.4) 16 (15.5)
**First-time provider,** ***n* (%)** Yes No	20 (100) 0	40 (100) 0	42 (97.7) 1 (2.3)	101 (98.1) 2 (1.9)
**Monthly household** **income (in SGD),** ***n* (%)** <2000 2000–4000 4000–8000 >8000 No income N/A	7 (35) 2 (10) 1 (5) 6 (30) 4 (20) 0	14 (35) 5 (12.5) 9 (22.5) 8 (20) 2 (5) 2 (5)	18 (41.86) 7 (16.28) 4 (9.3) 14 (32.56) 0 0	39 (37.9) 14 (13.6) 14 (13.6) 28 (27.2) 2 (1.9) 6 (5.8)

**Table 2:** Caregiver Patient Activation Measure (CG-PAM): overall scores and four activation levels by three included cancer diagnoses (*n* = 103)—lung, myeloma, and gastrointestinal (GI) cancer.

	Total Score, *n* (%), Median, ±STD ^1^	Lung, *n* (%), Median, ±STD	Myeloma, *n* (%), Median, ±STD	GI, *n* (%), Median, ±STD
**Level 1:** May not believe their role is important (≤47.0)	5 (4.9), 45.3, ±1.17	2 (4.65), 44.5, ±1.13	2 (1.9), 46.15, ±1.2	1 (0.1), 45.3
**Level 2:** Lacking knowledge and confidence to take action (≤47.1 and ≤55.1)	24 (23.3), 51, ±1.01	10 (23.26), 51, ±1.3	6 (5.8), 51, ±1.4	9 (8.7), 51, ±0.7
**Level 3:** Acting and feeling confident in gaining control (≥55.2 and ≤72.4)	42 (40.8), 60.6, ±5.0	20 (46,51), 60.6, ±5.5	9 (8.7), 60.6, ±4.6	12 (11.7), 61.9, ±4.7
**Level 4:** Confident but may struggle with maintaining behaviors over time (≥72.5)	32 (31.1), 82.85, ±10.5	11 (25.58), 80.9, ±10.3	3 (2.9), 100, ±12.9	18 (17.5), 84.8, ±10.6
**Overall score**	Median: 60.6, mean: 65.9, STD: ±16.1			

^1^. Standaard deviation (STD).

**Table 3:** Logistic regression of sociodemographic and characteristics of caregivers and caregiving activation.

Category	*p*-Value	Odds Ratio, 95% CI
Age	0.78	0.99 (0.97–1.03)
Education (lower versus higher)	0.87	1.08 (0.45–2.6)
Ethnicity (Chinese versus other)	0.35	0.65 (0.26–1.61)
Cancer diagnoses Lung versus myeloma GI versus myeloma	0.84 0.68	0.89 (0.28–2.8) 1.29 (0.39–4.24)
Caregiving length	0.58	1.4 (0.42–4.73)
Relation of caregiver to the patient (partner versus other)	0.42	1.42 (0.60–3.34)
Occupation (worker versus not worker)	0.26	0.60 (0.25–1.44)
Gender	0.29	1.63 (0.66–4.04)
Civil status (married versus single)	0.99	1.01 (0.39–2.54)
Monthly income (<4000 versus ≥4000 SGD)	0.57	0.43 (0.18–1.03)

## Data Availability

The data are not publicly available due to privacy and ethical restrictions. In accordance with the Singaporean ethics approval, the original dataset may be accessed only by members of the research team. However, the reported results in the manuscript include all collected data in fully anonymized form.

## Appendix A

**Table A1:** Distribution of responses to 13 self-management statements (CG-PAM).

Statements 1–13	Disagree Strongly, *n* (%)	Disagree, *n* (%)	Agree, *n* (%)	Agree Strongly, *n* (%)	N/A, *n* (%)
I am responsible for seeing that this persons health is managed properly.	0	3 (3)	28 (27)	72 (70)	0 (0.0)
Taking an active role in this person healthcare is one of the most important factors in determining her/his health and ability to function.	0	3 (3)	33 (32)	67 (65)	0 (0.0)
I am confident I can actions that will help prevent or minimize some symptoms or problems associated with this persons health.	0	4 (3.88)	57 (55.34)	35 (33.98)	7 (6.8)
I know what each of this persons prescribed medications does.	1 (1)	2 (2)	47 (46)	29 (28)	24 (23)
I am confident that I can tell when this person needs to get medical care and when I can handle the problem myself.	3 (3)	1 (1)	54 (52)	43 (42)	2 (2)
I am confident that I can tell a doctor or nurse concerns I have about this persons health even when he or she does not ask.	3 (2.91)	6 (5.83)	68 (66.02)	21 (20.39)	5 (4.85)
I am confident that I can carry out medical treatments I need to do for this person at home.	1 (0.97)	5 (4.85)	53 (51.46)	28 (27.19)	16 (15.53)
I understand the nature and causes of this persons health.	0	9 (9)	47 (46)	29 (28)	17 (17)
I know that different medical treatment options available for this persons health.	0	14 (13.6)	38 (36.9)	34 (33)	17 (16.5)
I am able to help this person maintain lifestyle changes, like healthy eating or exercising, for her/his condition.	0	8 (8)	65 (63)	27 (26)	3 (3)
I know how to prevent problems with this persons health.	2 (2)	5 (5)	68 (66)	26 (25)	2 (2)
I am confident I can work out solutions when new problems arise with this persons health.	6 (6)	8 (8)	63 (61)	23 (22)	3 (3)
I am confident I can help this person with lifestyle changes, like healthy eating and exercise, even during times of stress.	0	11 (11)	68 (66)	23 (22)	1 (1)
